# Influence of Fc Modifications and IgG Subclass on Biodistribution of Humanized Antibodies Targeting L1CAM

**DOI:** 10.2967/jnumed.121.262383

**Published:** 2022-04

**Authors:** Sai Kiran Sharma, Maya Suzuki, Hong Xu, Joshua A. Korsen, Zachary Samuels, Hongfen Guo, Brandon Nemieboka, Alessandra Piersigilli, Kimberly J. Edwards, Nai-Kong V. Cheung, Jason S. Lewis

**Affiliations:** 1 Department of Radiology, Memorial Sloan Kettering Cancer Center, New York, New York;; 2 Department of Pediatrics, Memorial Sloan Kettering Cancer Center, New York, New York;; 3 Center for Clinical and Translational Research, Kyushu University Hospital, Fukuoka, Japan;; 4 Molecular Pharmacology Program, Memorial Sloan Kettering Cancer Center, New York, New York;; 5 Department of Pharmacology, Weill Cornell Medical College, New York, New York;; 6 Gerstner Sloan Kettering Graduate School of Biomedical Sciences, Memorial Sloan Kettering Cancer Center, New York, New York;; 7 Tri-Institutional Laboratory of Comparative Pathology, Memorial Sloan Kettering Cancer Center, Weill Cornell Medical College, and Rockefeller University, New York, New York;; 8 Department of Radiology, Weill Cornell Medical College, New York, New York; and; 9 Radiochemistry and Molecular Imaging Probes Core, Memorial Sloan Kettering Cancer Center, New York, New York

**Keywords:** immuno-PET, aglycosylated antibody, afucosylated antibody, Fab arm exchange

## Abstract

Immuno-PET is a powerful tool to noninvasively characterize the in vivo biodistribution of engineered antibodies. **Methods:** L1 cell adhesion molecule–targeting humanized (HuE71) IgG_1_ and IgG_4_ antibodies bearing identical variable heavy- and light-chain sequences but different fragment crystallizable (Fc) portions were radiolabeled with ^89^Zr, and the in vivo biodistribution was studied in SKOV3 ovarian cancer xenografted nude mice. **Results:** In addition to showing uptake in L1 cell adhesion molecule–expressing SKOV3 tumors, as does its parental counterpart HuE71 IgG_1_, the afucosylated variant having enhanced Fc-receptor affinity showed high nonspecific uptake in lymph nodes. On the other hand, aglycosylated HuE71 IgG_1_ with abrogated Fc-receptor binding did not show lymphoid uptake. The use of the IgG_4_ subclass showed high nonspecific uptake in the kidneys, which was prevented by mutating serine at position 228 to proline in the hinge region of the IgG_4_ antibody to mitigate in vivo fragment antigen-binding arm exchange. **Conclusion:** Our findings highlight the influence of Fc modifications and the choice of IgG subclass on the in vivo biodistribution of antibodies and the potential outcomes thereof.

## Visual Abstract

**Figure:**
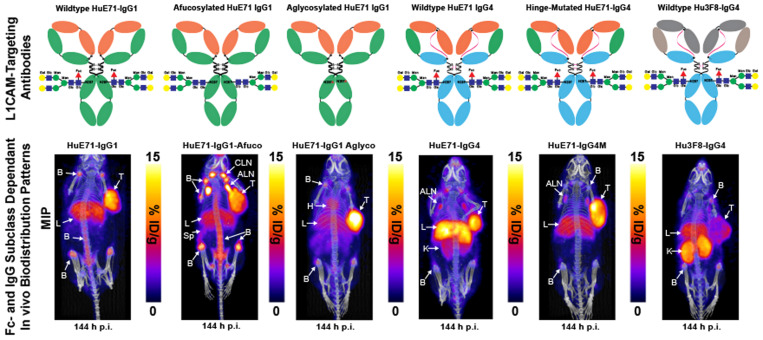

Monoclonal antibodies rank among the most sought-after class of pharmaceuticals being developed for the treatment of several diseases in humans (*1*). Their increasing utility has bolstered antibody-engineering efforts to improve efficacy and mitigate toxicities (*2*,*3*). Altering the glycosylation status, introducing point mutations in the fragment crystallizable (Fc) region, and changing the immunoglobulin G (IgG) subclass are common strategies whereby the binding of an IgG to Fc γ-receptors (FcγR) on immune effector cells can be modulated (*4*–*7*). However, the impact of these modifications on antibody biodistribution has not been adequately examined. Arguably, most therapeutic antibodies are unnaturally engineered biomolecules synthesized using recombinant technologies; hence, their in vivo biodistribution cannot be taken for granted. Intriguingly, of all the Food and Drug Administration (FDA)–approved antibodies, only a few have dynamic time-dependent in vivo biodistribution and pharmacokinetics data profiled in patients (*8*). Furthermore, only a handful of these antibodies have had preclinical biodistribution analysis before or after FDA approval (*9*). Longitudinal imaging by immuno-PET can fill this existing knowledge gap by enabling quantitation of the in vivo pharmacokinetics and biodistribution of antibodies while delineating their on-target binding and off-target disposition. Critically, immuno-PET and biodistribution studies performed in relevant preclinical animal models early in antibody drug development campaigns can serve as a harbinger for clinical translation and success of antibody therapeutics in human patients (*10*,*11*).

Most FDA-approved antibody therapeutics belong to the fully human or humanized IgG_1_ subclass. In addition to target-specific binding at the fragment antigen-binding end of the IgG molecule, human or humanized IgG_1_ antibodies bind strongly to activating FcγRs such as FcγRIIIa, which is expressed on immune effector cells such as natural killer cells to mediate antibody-dependent cellular cytotoxicity (ADCC), a key mechanism of action of several therapeutic antibodies. Furthermore, afucosylated IgG_1_ antibodies lacking a core fucose in the N-linked biantennary oligosaccharide units of the Fc region have stronger Fc–FcγRIIIa binding, leading to enhanced ADCC activity (Fig. 1) (*12*). On the other hand, aglycosylated IgG_1_ antibodies lacking the N-linked biantennary oligosaccharide unit in the Fc region have abrogated Fc–FcγR interactions (Fig. 1) (*13*). Of late, IgG_4_—the least abundant IgG in human serum—has emerged as a subclass of choice for the development of therapeutic antibodies, including those used for immunotherapy (*14*). The low affinity of IgG_4_ antibodies for activating FcγRs but high affinity for the inhibitory FcγRIIB renders them relatively benign for ADCC. In fact, IgG_4_s are considered antiinflammatory antibodies because of their ability to dampen immune responses against repetitive allergen exposure (*15*). These properties make IgG_4_ a subclass of choice for the design of immunotherapeutics such as nivolumab and pembrolizumab, which bind to programmed cell death protein 1 on effector T cells in the tumor microenvironment and render efficacy without eliciting secondary immune mechanisms such as ADCC (*5*,*7*,*16*). Collectively, all the aforementioned features highlight the importance of in vivo biomolecular interactions along the Fc–Fc receptor axis that are worth considering during the design and development of therapeutic antibodies (*17*).

**FIGURE 1.**
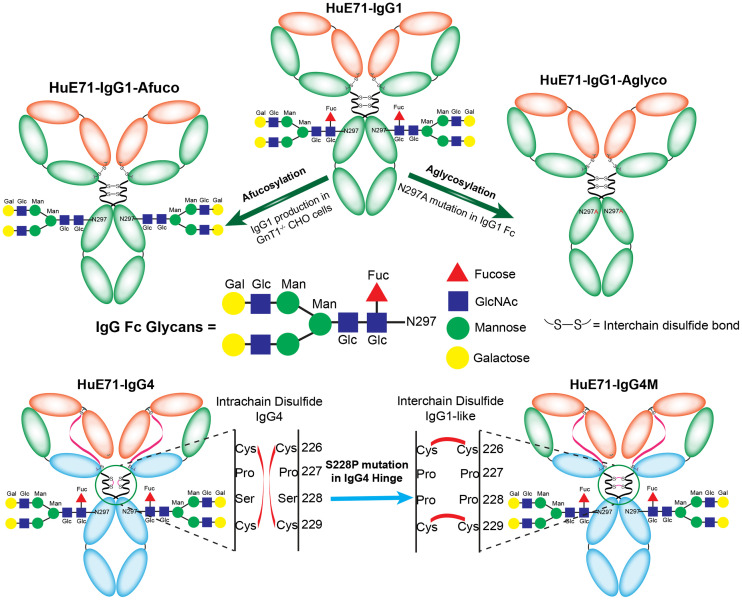
Schematic describing the generation of Fc variants of humanized IgG_1_ and hinge-mutated IgG_4_ L1CAM-targeted antibodies. Fc-glycosylated variants of L1CAM-targeted humanized IgG_1_ antibody, HuE71-IgG_1_ (top center), were obtained by producing IgG_1_ antibody in GnT1^−/−^ CHO cells that are defective for fucosylation and thus yield HuE71-IgG_1_-Afuco (top left) (25), whereas substituting asparagine at position 297 in Fc region to alanine (N297A) yielded aglycosylated variant, HuE71-IgG_1_-Aglyco (top right). Engrafting anti-L1CAM binding variable heavy- and variable light-chain sequences onto IgG_4_ framework yielded HuE71-IgG_4_ (bottom left). S228P in hinge region of HuE71-IgG_4_ yielded HuE71-IgG_4_M. Fuc = fucose; Glc = *N*-acetylglucosamine; Man = mannose; Gal = *N*-acetylgalactosamine.

In the work at hand, we asked 3 questions fundamental to the molecular composition of humanized IgGs targeting the cell surface glycoprotein L1 cell adhesion molecule (L1CAM), without interfering with the antibody’s ability to bind its cognate antigen or interact with the neonatal Fc receptor. Foremost, we asked how enhancement of Fc–FcγR affinity by afucosylation impacts the in vivo distribution of humanized IgG_1_. Next, we were curious to know how Fc silencing via antibody aglycosylation, which abrogates Fc–FcγR interaction, influences the in vivo distribution of humanized IgG_1_. Lastly, we wanted to know how the choice of IgG subclass—switching from IgG_1_ to IgG_4_ with and without fab arm exchange (FAE), and loss of most Fc functions—affects antibody distribution in vivo. To that end, we developed a panel of humanized antibodies (Fig. 1; Table 1) targeting human L1CAM, which is overexpressed in several malignancies (*18*,*19*). To noninvasively visualize the antibodies in vivo, we radiolabeled them with ^89^Zr and used immuno-PET in athymic nude mice bearing subcutaneously implanted L1CAM-expressing SKOV3 tumors.

**TABLE 1.** Antibodies Used in This Study and Their Biochemical and Functional Characteristics

Antibody	HuE71-IgG_1_	HuE71-IgG_1_ Afucosylated	HuE71-IgG_1_ Aglycosylated	HuE71-IgG_4_	HuE71-IgG_4_M	Hu3F8-IgG_4_
Target	L1CAM	L1CAM	L1CAM	L1CAM	L1CAM	GD2
Immunoreactive fraction (%)	93.1 ± 2.2	89.5 ± 1.5	85.8 ± 2.9	86.7 ± 0.2	88.6 ± 0.3	NA
Subclass	IgG_1_	IgG_1_	IgG_1_	IgG_4_	IgG_4_	IgG_4_
Antibody modification	Wild-type IgG_1_	Afucosylated IgG_1_	Aglycosylated IgG_1_	Wild-type IgG_4_	S228P Mut IgG_4_	Wild-type IgG_4_
FcγR binding	++	+++	−	+	+	+
Fragment antigen-binding arm exchange	−	−	−	+	−	+

## MATERIALS AND METHODS

### Animal Model

All animals were treated as per guidelines approved by the Research Animal Resource Center and Institutional Animal Care and Use Committee at Memorial Sloan Kettering Cancer Center. Female athymic nude (Nu/Nu) mice 8–10 wk old were purchased from Charles River Laboratories. Animals were housed in ventilated cages, given food and water ad libitum, and allowed to acclimatize for 1 wk before inoculation of tumor cells. SKOV3 tumors were induced on the right shoulder via subcutaneous injection of 5 million cells in a 150-μL cell suspension of a 1:1 (v/v) mixture of fresh medium and Matrigel (BD Biosciences). The xenografted mice were used for in vivo studies when the tumor volumes reached approximately 300 mm^3^.

### PET Imaging

PET imaging was conducted using a mouse hotel on an Inveon PET/CT scanner (Siemens Healthcare) (*20*). SKOV3-xenografted mice were intravenously administered ^89^Zr-labeled antibodies (8 MBq; 45 μg suspended in 150 μL of phosphate-buffered saline per mouse; *n* = 2 mice per antibody variant). Animals were scanned under the influence of anesthesia by inhalation of 2% isoflurane (Baxter Healthcare) and medical air. PET data for each mouse were recorded via static scans at 48, 96, and 144 h after injection. The PET/CT images were calibrated and cropped before analysis and scaled using AMIDE software (Stanford University). The images were rendered using VivoQuant (Invicro).

### Biodistribution

Ex vivo biodistribution analysis was performed on a separate cohort of SKOV3-xenografted mice that were intravenously administered 1.15 MBq (6.4 μg of each ^89^Zr-labeled antibody variant suspended in 150 μL of phosphate-buffered saline per mouse). Six animals were used per antibody variant, wherein 3 animals were injected with ^89^Zr-labeled antibody alone and 3 animals were injected with a mixture of ^89^Zr-labeled antibody and a 38-fold excess (mass) of the unlabeled antibody variant. Animals were euthanized by CO_2_ asphyxiation at 144 h after injection. After euthanasia, tissues of interest were harvested via necropsy, weighed, and assayed for radioactivity on a γ-counter calibrated for ^89^Zr. Counts were converted into activity using a calibration curve generated from known standards. Count data were background- and decay-corrected to the time of injection, and the percentage injected dose (%ID) per gram for each tissue sample was calculated by normalization to the total activity injected.

### Statistics

All data are expressed as mean ± SD. Statistical analysis was performed using GraphPad Prism, version 9.1.0. Statistical comparisons of radioactivity concentrations in each organ across the various groups in the ex vivo biodistribution studies were done using nonparametric multiple Mann–Whitney tests to compare ranks. The Holm–Šídák multiple-comparison test was applied, and the threshold for *P* value comparison was set to 0.05.

## RESULTS

A panel of IgG_1_ and IgG_4_ antibodies having identical variable heavy- and light-chain sequences targeting human L1CAM but modified Fc regions was generated (Table 1) to gain insights into the influence of Fc modifications and subclass on the in vivo biodistribution of IgG_1_ and IgG_4_ antibodies, respectively.

After purification of the various ^89^Zr-labeled antibodies, we obtained radioimmunoconjugates having an average molar activity of 26.6 MBq/nmol. A cell-based immunoreactivity assay confirmed the ability of the various radioimmunoconjugates to bind L1CAM-expressing SKOV3 cells (Table 1; Supplemental Fig. 1; supplemental materials are available at http://jnm.snmjournals.org) (*21*). Incubation of the radioimmunoconjugates in serum and evaluation by radio–instant thin-layer chromatography demonstrated less than 4% demetallation up to 7 d after radiosynthesis, suggesting high stability of the radioimmunoconjugates in a biologically relevant medium (Supplemental Fig. 2). Size-exclusion high-performance liquid chromatography of the ^89^Zr-labeled antibodies incubated without a radioprotectant in chelexed phosphate-buffered saline at 37°C showed more than 80% of the radioimmunoconjugates being stable and existing as monomers up to 6 d after radiosynthesis (Supplemental Figs. 3 and 4).

**FIGURE 2.**
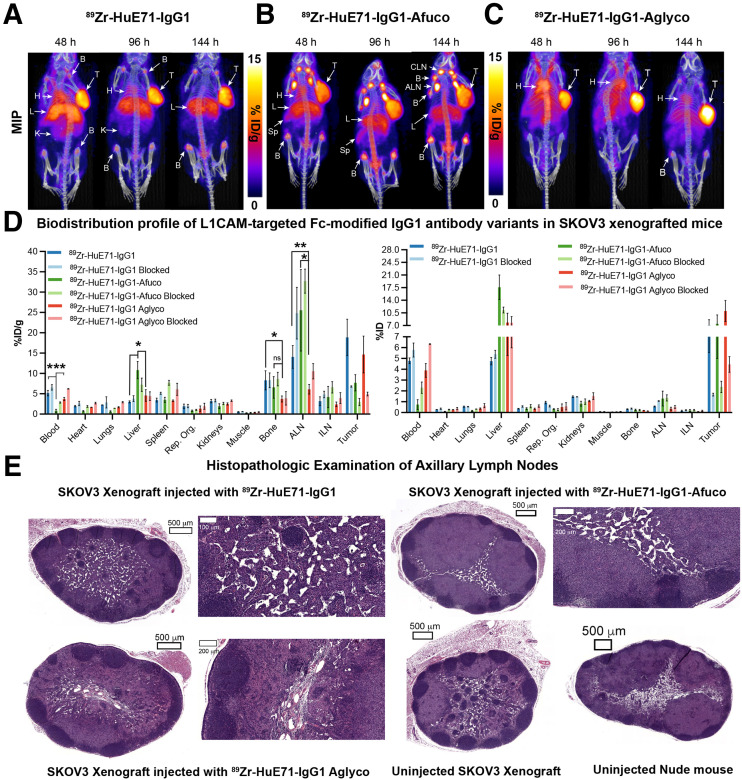
Delineation of differential in vivo profiles of Fc-modified L1CAM-targeted IgG_1_ variants in SKOV3-xenografted mice through immuno-PET imaging, ex vivo biodistribution analysis, and histopathology. (A–C) Longitudinal PET/CT images acquired at 48, 96, and 144 h after injection of 1.8 mg/kg (7.95 MBq, 45 μg) of ^89^Zr-HuE71-IgG_1_ (A), ^89^Zr-HuE71-IgG_1_-Afuco (B), and ^89^Zr-HuE71-IgG_1_-Aglyco (C) show distribution of radioactivity in blood (indicated by heart (H), tumor (T), liver (L), long bone joints, axillary lymph nodes (ALN), cervical lymph nodes (CLN), and spleen (Sp). Maximum-intensity projections (MIPs) were calibrated and scaled 0–15 %ID/g. (D) Ex vivo biodistribution profile (%ID/g vs. %ID) at 144 h after injection of 0.25 mg/kg (1.15 MBq, 6.4 μg) of 3 ^89^Zr-labeled L1CAM-targeted Fc-modified IgG_1_ variants and their corresponding low-specific-activity blocking dose groups in SKOV3-xenografted mice. Detailed % ID/g and %ID values can be found in Supplemental Tables 1 and 2. (E) Panel of representative low- and high-magnification hematoxylin- and eosin-stained images from histopathologic examination of axillary lymph nodes harvested from SKOV3-xenografted mice injected with the three ^89^Zr-labeled L1CAM-targeted IgG_1_ Fc variants compared with low-magnification images of axillary lymph nodes harvested from uninjected SKOV3-xenografted mouse and healthy tumor-naïve nude mouse. Scale bars on low-magnification images represent 500 μm, whereas those on high-magnification images represent 200 μm. ILN = inguinal lymph node; *P ≤ 0.03. **P ≤ 0.01. ***P ≤ 0.0005.

Athymic nude mice were used in our studies because of their ability to grow tumors from implanted human cancer cell lines and the presence of functional innate immune cells such as macrophages, dendritic cells, and natural killer cells in this strain. Macrophages and natural killer cells comprise 2 main Fc-dependent effector cells responsible for eliciting antibody-dependent cellular phagocytosis and ADCC, respectively (*22*). Furthermore, despite only 60%–70% homology between mouse and human FcγRs, human IgGs are reported to bind orthologous mouse FcγRs with similar strength, suggestive of potentially similar downstream biologic activities mediated by human Fc–murine FcγR interactions in mice (*23*). Immuno-PET imaging of the three ^89^Zr-labeled IgG_1_ variants—humanized (HuE71)-IgG_1_, HuE71-IgG_1_-Afuco, and HuE71-IgG_1_-Aglyco—demonstrated uptake of radioactivity in SKOV3 tumors (Figs. 2A–2C). However, the three IgG_1_ variants yielded distinct vivo distribution patterns of radioactivity. SKOV3-xenografted mice injected with ^89^Zr-HuE71-IgG_1_ showed persistence of radioactivity in blood up to 96 h after injection, suggesting slow in vivo clearance of L1CAM-targeted antibodies in this model (Fig. 2A). Besides target-specific tumoral uptake of radioactivity, nonspecific uptake was found in the liver and joints of the long bones of mice. Similarly, ^89^Zr-HuE71-IgG_1_-Afuco yielded uptake of radioactivity in the tumor, liver, and joints of the long bones. However, this variant revealed high-intensity bilateral hot spots corresponding to the axillary and cervical lymph nodes (Fig. 2B) and demonstrated increased clearance from circulation relative to ^89^Zr-HuE71-IgG_1_. Additionally, ^89^Zr-HuE71-IgG_1_-Afuco outlined the spleen and showed a higher radioactivity concentration in long bone joints and the vertebral column. The faster clearance and elevated nonspecific tissue uptake patterns observed for ^89^Zr-HuE71-IgG_1_-Afuco may be attributed to enhanced binding of the afucosylated Fc with mouse FcγRIV-expressing immune cells in the lymph nodes and reticuloendothelial system. The latter is exemplified by results from the analysis of Fc–FcγR binding by surface plasmon resonance (Table 2). Notably, conjugation of desferrioxamine to lysine residues distributed randomly in the Fc region of IgG_1_ molecules did not impact binding to murine FcγRIV and human FcγRIIIa-158V.

**TABLE 2.** Analysis of Fc–Fc Receptor Binding by Surface Plasmon Resonance (Biacore T200)

Antibody	KD (M) murine FcγRIV	Relative murine FcγRIV binding	KD (M) human FcγRIIIa-158V	Relative human FcγRIIIa-158V binding
HuE71-IgG_1_	7.24E−07	1.00	8.73E−07	1.00
Desferrioxamine-HuE71-IgG_1_	5.39E−07	1.34	6.64E−07	1.31
HuE71-IgG_1_-Afuco	2.27E−07	3.19	2.86E−07	3.05
Desferrioxamine-HuE71- IgG_1_-Afuco	2.19E−07	3.31	2.94E−07	2.96
HuE71-IgG_1_-Aglyco	NB	—	NB	—
Desferrioxamine-HuE71- IgG_1_-Aglyco	NB	—	NB	—

KD (M) = equilibrium dissociation constant; NB = no binding.

Previous studies found significant increases in the binding affinity of afucosylated IgG_1_ antibody to human FcγRIIIa but no change in the binding affinity to human FcγRI and human neonatal Fc receptor (*12*,*24*,*25*). So, we focused our surface plasmon resonance (SPR) analysis of the differentially glycosylated IgG_1_ Fc variants to human FcγRIIIa. Murine FcγRIV was included in the surface plasmon resonance assay since it is a functional ortholog of human FcγRIIIa, and binding to murine FcγRIV may contextualize findings from in vivo studies performed in mice (*26*). Notably, ADCC in humans is mediated via interaction of the Fc of target antigen-bound hIgG_1_ and human FcγRIIIa expressed on immune effector cells. Furthermore, afucosylated human or humanized IgG_1_ antibodies have been shown to target murine FcγRIV for enhanced tumor therapy by ADCC in mice (*27*). SPR analysis of the anti-L1CAM IgG_1_ variants used in our study demonstrated 3-fold higher affinity of the HuE71-IgG_1_-Afuco for murine FcγRIV than of the HuE71-IgG_1_. Interestingly, HuE71-IgG_1_-Afuco also showed a similar 3-fold higher binding to the 158V isoform of human FcγRIIIa. On the other hand, and as expected, HuE71-IgG_1_-Aglyco showed no binding to either mouse or human FcγRs. Lastly, immuno-PET of ^89^Zr-HuE71-IgG_1_-Aglyco in SKOV3-xenografted mice displayed tumoral uptake of radioactivity with a relatively lower concentration in the liver and bone joints and no detectable uptake in lymph nodes (Fig. 2C). ^89^Zr-HuE71-IgG_1_-Aglyco demonstrated the longest persistence of radioactivity in systemic circulation, suggesting an enhanced in vivo half-life plausibly due to the lack of human Fc–murine FcγRIV interactions with resident immune effector cells in the lymph nodes and reticuloendothelial system.

Quantification of the in vivo biodistribution of the three L1CAM-targeted IgG_1_ variants was done in a separate cohort of SKOV3 xenografted mice injected with the ^89^Zr-labeled anti-L1CAM-targeted Fc-modified antibodies. Foremost, the three IgG_1_ variants displayed differential radioactivity concentrations in the blood. ^89^Zr-HuE71-IgG_1_-Afuco showed less than 1 %ID/g remaining in circulation at 144 h after injection, whereas the other two variants showed up to 6 %ID/g at this time point. Next, ^89^Zr-HuE71-IgG_1_-Afuco demonstrated high liver uptake (10.8 ± 2.1 %ID/g) compared with the other two variants, which showed less than 6 %ID/g in this tissue. Most other nontarget tissues showed unremarkable differences in uptake of radioactivity between the three Fc-modified IgG_1_ variants. However, axillary lymph nodes isolated from SKOV3-xenografted mice injected with ^89^Zr-HuE71-IgG_1_-Aglyco yielded a significantly lower radioactivity concentration in this tissue. Unlike PET images, only mice injected with ^89^Zr-HuE71-IgG_1_ demonstrated a significantly higher radioactivity concentration in the bone (femur) than did xenografts injected with ^89^Zr-HuE71-IgG_1_-Aglyco. Indeed, SKOV3 tumors showed high and specific uptake of radioactivity for all three ^89^Zr-labeled L1CAM-targeted IgG_1_ variants. However, the tumoral uptake values (%ID/g) in mice dosed with the unblocked L1CAM-targeted ^89^Zr-radioimmunoconjugates demonstrated significantly decreased uptake of ^89^Zr-HuE71-IgG_1_-Afuco compared with radioimmunoconjugates of the other two IgG_1_ variants. The relatively low tumoral uptake (7.7 ± 2 %ID/g) of ^89^Zr-HuE71-IgG_1_-Afuco may be attributed to concentration of a significant proportion of the radioactivity or antibody in the liver and lymph nodes of SKOV3-xenografted mice. Determining the %ID taken up in the various tissues revealed that despite having the highest radioactivity concentration (%ID/g) for ^89^Zr-HuE71-IgG_1_-Afuco, the axillary lymph nodes had less than 2% of the total injected radioactivity at 144 h after injection. Instead, the liver accumulated more radioactivity (17.6 ± 3.4 %ID) and turned out to be a major sink for the afucosylated IgG_1_ variant.

Importantly, the histopathologic examination of lymph nodes harvested from SKOV3-xenografted mice injected with ^89^Zr-HuE71-IgG_1_-Afuco showed no morphologic evidence of infiltrating neoplastic cells. Instead, these nodes demonstrated reactive hyperplasia characterized by marked paracortical and medullary histio- and plasmacytosis (Fig. 2E). The latter was a unique feature relative to lymph nodes harvested from SKOV3-xenografted mice and tumor-naïve mice that never received ^89^Zr-HuE71-IgG_1_-Afuco. Along those lines, axillary lymph nodes harvested from SKOV3-xenografted mice injected with ^89^Zr-HuE71-IgG_1_ and ^89^Zr-HuE71-IgG_1_-Aglyco showed minor sinus histiocytosis but displayed normal lymphoid tissue architecture (Fig. 2E).

**FIGURE 3.**
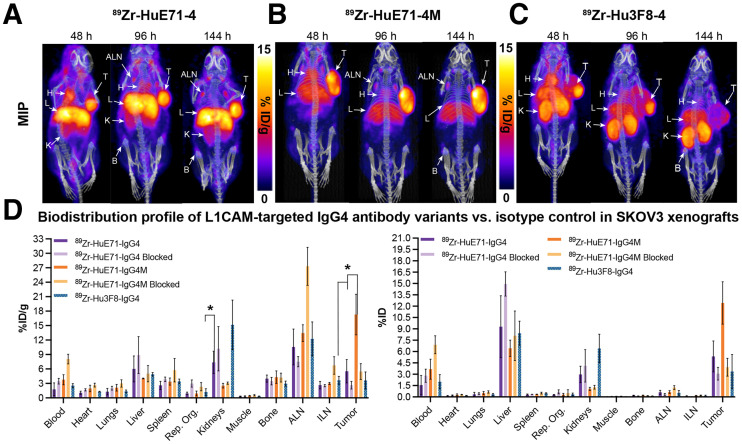
Delineation of differential in vivo profiles of L1CAM-targeted IgG_4_ variants in SKOV3-xenografted mice through immuno-PET imaging and ex vivo biodistribution analysis. (A–C) Longitudinal PET/CT images acquired at 48, 96, and 144 h after injection of 1.8 mg/kg (7.95 MBq, 45 μg) of ^89^Zr-HuE71-IgG_4_ (A), ^89^Zr-HuE71-IgG_4_M (B), and ^89^Zr-Hu3F8-IgG_4_ (C) show distribution of radioactivity in blood (indicated by heart [H]), tumor (T), liver (L), kidneys (K), axillary lymph nodes (ALN), and long bone joints (B). Maximum intensity projections (MIPs) were calibrated and scaled 0%–15 %ID/g. (D) Ex vivo biodistribution profile (%ID/g vs. %ID) at 144 h after injection of 0.25 mg/kg (1.15 MBq, 6.4 μg) of 2 ^89^Zr-labeled L1CAM-targeted IgG_4_ variants and isotype control IgG_4_ antibody in SKOV3-xenografted mice. ILN = inguinal lymph node. *P ≤ 0.05. Detailed % ID/g and %ID values can be found in Supplemental Tables 3 and 4.

Next, we studied the influence of IgG subclass on the in vivo biodistribution of antibody drugs. To delineate the in vivo biodistribution of IgG_4_ antibodies, we generated a humanized IgG_4_ variant of the L1CAM-targeting antibody and conducted serial PET imaging studies in SKOV3-xenografted mice. Serial PET imaging of ^89^Zr-HuE71-IgG_4_ revealed slow in vivo clearance of the antibody from blood and a high radioactivity concentration in the liver (Fig. 3A). In addition to tumoral uptake of radioactivity, ^89^Zr-HuE71-IgG_4_ showed a radioactivity concentration in the kidneys and axillary lymph nodes of SKOV3 xenografts. We hypothesized that uptake of radioactivity in the kidneys may be attributed to FAE, leading to instability of the antibody in vivo. FAE is an intrinsic property of the IgG_4_ subclass whereby two half-molecules (heavy-chain–light-chain pair) of the antibodies dissociate from one another at the hinge and recombine spontaneously with other IgG_4_ half-molecules in serum to form monovalent bispecific antibodies in vitro and in vivo (*28*). Introducing a point mutation from serine to proline at position 228 (S228P) in the hinge region of IgG_4_ antibodies has been shown to mitigate the propensity of FAE (*6*). To validate our hypothesis, an S228P hinge-mutated IgG_4_ variant—^89^Zr-HuE71-IgG_4_M—was synthesized and evaluated in vivo. ^89^Zr-HuE71-IgG_4_M demonstrated gradual accretion of radioactivity in the SKOV3 tumor while showing little to no radioactivity in the kidneys (Fig. 3B). Of note, ^89^Zr-HuE71-IgG_4_M faintly highlighted the liver, axillary lymph nodes, and bone joints in this model.

The use of an IgG_4_ variant of the anti-GD2 antibody, Hu3F8, as an isotype control and the similarity in radioactivity uptake in the kidneys of SKOV3-xenografted mice injected with ^89^Zr-Hu3F8-IgG_4_ and ^89^Zr-HuE71-IgG_4_ further validated that the abnormal kidney uptake is attributed to FAE intrinsic to IgG_4_ antibodies (Fig. 3C). Further, results from an ex vivo biodistribution analysis performed on SKOV3-xenografted mice injected with the three ^89^Zr-labeled IgG_4_ antibodies corroborated findings from immuno-PET studies (Fig. 3D). Importantly, ^89^Zr-HuE71-IgG_4_M yielded a significantly lower radioactivity concentration (2.5 ± 0.56 %ID/g) in the kidneys than did ^89^Zr-HuE71-IgG_4_ (7.4 ± 2.32 %ID/g; *P* = 0.02). Furthermore, blockade of tumoral uptake of radioactivity by coinjection of a 0.25 mg/kg dose of ^89^Zr-labeled IgG_4_ antibody with a 38-fold excess (mass) of unmodified L1CAM-targeted IgG_4_ antibodies in ex vivo biodistribution studies confirmed target-mediated uptake in SKOV3 tumors (Fig. 3D). Notably, ^89^Zr-HuE71-IgG_4_M demonstrated increased tumoral uptake of radioactivity (16.1 ± 4.26 %ID/g) compared with ^89^Zr-HuE71-IgG_4_ (5.5 ± 2.4 %ID/g; *P* = 0.03) and ^89^Zr-Hu3F8-IgG_4_ (3.6 ± 1.76 %ID/g; *P* = 0.02). Concordant with PET data, ^89^Zr-Hu3F8-IgG_4_ yielded a high radioactivity concentration in the kidneys (15.2 ± 5.14 %ID/g) and showed between 1 and 8 %ID/g in most healthy tissues. Tumoral uptake (4.9 ± 0.46 %ID/g) of the isotype antibody may be attributed to enhanced permeability and retention in this compartment. Lastly, the high radioactivity concentration in multiple tissues harvested from mice injected with the ^89^Zr-HuE71-IgG_4_M-blocking dose arm is most likely a result of persistence of ^89^Zr-HuE71-IgG_4_M in the blood at 144 h after injection.

## DISCUSSION

Recent insights into pharmacologic modulation at the Fc–FcγR axis have made this molecular interaction an important consideration in the development of antibody-based drugs for cancer immunotherapy (*16*,*17*,*29*). Furthermore, single-nucleotide polymorphisms in FcγR-encoding genes have been implicated in disease etiology and clinical responses (*30*). Specifically, patients carrying the 158V/V genotype showed improved outcomes from rituximab therapy due, in part, to improved ADCC activity in vivo (*31*). Additionally, we have previously shown that stronger in vitro Fc–FcγR binding for an afucosylated variant of the humanized anti-GD2 IgG_1_ antibody yielded improved preclinical efficacy because of enhanced ADCC in vivo (*25*).

Our current findings with the afucosylated anti-L1CAM IgG_1_ variant concur broadly with two ^89^Zr-immuno-PET studies done using HER3-targeted humanized IgG_1_ antibodies—GSK2849330 and RG7116—which were Fc-glycoengineered for enhanced ADCC activity (*32*,*33*). The high uptake of radioactivity in the liver and spleen of xenograft models developed in immunodeficient SCID mice used in those studies was attributed to enhanced binding of the antibodies with FcγRs expressed on tissue-resident auxiliary immune cells in the reticuloendothelial system (*32*,*33*). However, neither of those antibodies showed elevated radioactivity concentrations in lymph nodes. The latter may be due, in part, to the higher immunodeficient status of SCID mice used in those studies and the presence of functional natural killer cells in athymic nude mice used in our study. Although suggestive of an Fc-mediated phenomenon, the pronounced lymph node uptake of radioactivity in mice injected with the afucosylated variant warrants further validation in immunocompetent syngeneic tumor models or xenograft models developed in mice reconstituted with a functional human immune system. Admittedly, immunodeficient mice impact the in vivo biodistribution of exogenously injected human or humanized IgG_1_ because of relatively low titers of endogenous IgG and the availability of unoccupied high-affinity FcγRs on tissue-resident immune cells in the liver, spleen, and bone marrow (*34*). This phenomenon is exacerbated in highly immunodeficient mouse strains developed on the NOD-SCID background (*35*). However, low levels of serum IgG_2a_ in athymic nude mice have also been implicated in the rapid clearance of exogenously injected human IgG_1_ and mouse IgG_2a_ (*36*).

Along those lines, a comparison of the afucosylated versus parental L1CAM-targeting IgG_1_ in tumor-naïve athymic nude mice revealed a lower radioactivity concentration of ^89^Zr-HuE71-IgG_1_-Afuco in the blood at 120 h after injection, suggesting faster in vivo pharmacokinetics (Supplemental Fig. 5), which is consistent with our findings for this variant in SKOV3-xenografted mice. Furthermore, the relatively high radioactivity concentration in the long bone joints of mice injected with ^89^Zr-HuE71-IgG_1_-Afuco is also indicative of faster in vivo catabolism of the radioimmunoconjugate, leading to the release of ^89^Zr for in vivo uptake and complexation with hydroxyapatite in the bone joints. When target expression is absent in the bones, radioactivity uptake in this tissue is commonly attributed to the in vivo catabolism of desferrioxamine-conjugated ^89^Zr-labeled antibodies in mice and the osteophilic nature of ^89^Zr (*37*). Intriguingly, there was no significant difference between radioactivity concentrations in the liver and those in the axillary lymph nodes harvested from tumor-naïve mice injected with either ^89^Zr-labeled L1CAM-targeted IgG_1_ variant. That finding points to a potential contribution of the tumor—a target sink, which may account for a pronounced difference noted between ^89^Zr-HuE71-IgG_1_ and ^89^Zr-HuE71-IgG_1_-Afuco in SKOV3-xenografted animals. Taken together, our findings suggest that afucosylated IgG_1_ antibodies having improved Fc–FcγR binding and enhanced ADCC capability are likely to have significantly faster in vivo pharmacokinetics due to sequestration in the reticuloendothelial system and resident immune cells in lymph nodes. On the other hand, IgG_1_ aglycosylation yielded an Fc-silenced antibody, which showed no lymph node uptake when tested in the same animal model as its Fc-active counterparts.

In light of our findings with the L1CAM-targeting IgG_4_ variants, it is no surprise that several FDA-approved IgG_4_ antibody therapeutics harbor the S228P mutation to impart in vivo stability while minimizing therapeutic variability due to in vivo FAE (*14*). Of interest, lower uptake of radioactivity was found in the bone joints of SKOV3-xenografted mice injected with L1CAM-targeted ^89^Zr-labeled IgG_4_ antibodies than with their Fc-active IgG_1_ counterparts. Case in point, femurs harvested from SKOV3-xenografted mice injected with ^89^Zr-HuE71-IgG_4_ and ^89^Zr-HuE71-IgG_4_M showed 4.0 ± 0.74 %ID/g (*P* = 0.03) and 4.4 ± 1.58 %ID/g (*P* = 0.047), respectively, compared with ^89^Zr-HuE71-IgG_1_, which yielded 8.3 ± 2.35 %ID/g in this tissue. Furthermore, radioactivity concentrations of ^89^Zr-labeled L1CAM-targeted IgG_4_ variants were comparable to that yielded by the Fc-silent IgG_1_ variant—^89^Zr-HuE71-IgG_1_-Aglyco (3.7 ± 0.75 %ID/g)—in this tissue. The latter is suggestive of slow in vivo catabolism and low nonspecific uptake in healthy nontarget tissue. Lastly, the nonspecific hepatic uptake of radioactivity highlights a plausible contribution of Fc–FcγR interactions between Fc-active radiolabeled IgG_1_s and parenchymal and nonparenchymal cells in the liver (*38*). It is known that the liver is involved in the vivo catabolism of radiometal-labeled antibodies, leading to initial accumulation of ^89^Zr-radiometabolites and subsequent complexation of free ^89^Zr in the long bone joints of mice (*37*,*39*,*40*).

Highlights aside, a limitation of the current work is that it uses antibody variants developed for a single tumor-associated antigen in a singular xenograft model developed on an immunodeficient background. Additionally, identification of cells having elevated expression of murine FcγRIV in lymph nodes leading to the manifestation of reactive hyperplasia, and pinpointing cells in the liver that bind ADCC-enhanced IgG_1_ antibodies to impact in vivo pharmacokinetics, are outstanding questions that warrant further investigation.

## CONCLUSION

Collectively, our findings highlight the influence of Fc-glycosylation status and choice of IgG subclass on the in vivo biodistribution of the most widely used human or humanized antibody subclasses (IgG_1_ and IgG_4_) approved as therapeutics for human use. Our results demonstrate that deglycosylated IgG_1_ antibodies yield low nonspecific off-target uptake in healthy tissues, whereas S228P hinge-mutated IgG_4_ antibody eliminates FAE-mediated renal uptake of radioactivity. Importantly, this work illustrates the value of immuno-PET in delineating the in vivo biodistribution of ADCC-enhanced IgG_1_ antibodies and in macroscopically highlighting potential nontumor tissue depots. Doing so can inform antibody drug development efforts to uncover mechanisms leading to in vivo therapeutic benefit or toxicity. From a theranostic perspective, our results suggest that developing immuno-PET agents using ADCC-enhanced tumor-targeting IgG_1_ antibodies may yield false-positive results in lymph nodes because of Fc–FcγR interactions in vivo. Similarly, immuno-PET agents developed using tumor-targeting wild-type IgG_4_ antibodies may yield false-positive results from nonspecific uptake of radioactivity in the kidneys while grossly underestimating tumor burden because of loss of the radiotracer to in vivo FAE. In sum, we hope that the results described herein further motivate the use of molecular imaging to inform the preclinical development of novel antibody-based theranostic agents.

## DISCLOSURE

This work was supported by the MSKCC Small Animal Imaging Core, funded in part by NIH Small-Animal Imaging Research Program (SAIRP) grant R24 CA83084 and NIH center grant P30 CA08748 and by the Tri-Institutional Laboratory of Comparative Pathology, which was funded in part by NIH grant P30 CA08748. The work was also supported by an R01 CA176671 awarded to Jason Lewis and a diversity supplement R01 CA176671 awarded to Brandon Nemieboka. Brandon Nemieboka was supported by a Medical Scientist Training Program grant from the National Institute of General Medical Sciences of the National Institutes of Health under award number T32GM007739 to the Weill Cornell/Rockefeller/Sloan Kettering Tri-Institutional MD-PhD Program. Jason Lewis was supported by R01 CA204167 and R35CA232130. Jason Lewis acknowledges funding support from the Mr. William H. Goodwin and Mrs. Alice Goodwin and the Commonwealth Foundation for Cancer Research and the Center for Experimental Therapeutics at Memorial Sloan Kettering Cancer Center. Sai Kiran Sharma acknowledges the Tow Foundation for a Postdoctoral Fellowship Award. Nai-Kong Cheung, Maya Suzuki, Brandon Nemieboka, Hong Xu, and Jason Lewis were named as inventors on the L1CAM patent (WO2018232188A1) filed by Memorial Sloan Kettering. Both Memorial Sloan Kettering and Nai-Kong Cheung have financial interest in Y-mAbs Therapeutics Inc., Abpro-Labs, and Eureka Therapeutics. Nai-Kong Cheung reports receiving commercial research grants from Y-mAbs Therapeutics Inc. and Abpro-Labs Inc. Nai-Kong Cheung was named as inventor on multiple patents filed by Memorial Sloan Kettering, including those licensed to Y-mAbs Therapeutics Inc., Biotec Pharmacon, and Abpro-labs. Nai-Kong Cheung is a Scientific Advisory Board member for Abpro-Labs and Eureka Therapeutics. Jason Lewis is an associate editor of *The Journal of Nuclear Medicine* but had no involvement or access to information regarding the peer review of this article. Sai Kiran Sharma is a member of the editorial board of *The Journal of Nuclear Medicine.* No other potential conflict of interest relevant to this article was reported.

**KEY POINTS**

**QUESTION:** What is the impact of Fc modification and choice of IgG subclass on the in vivo pharmacologic profile of humanized antitumor antibodies?

**PERTINENT FINDINGS:** Humanized IgG_1_ antibodies yield differential in vivo pharmacokinetics and biodistribution based on the glycosylation status of the Fc. Afucosylated IgG_1_ antibodies with enhanced Fc–FcγR binding and ADCC activity yield faster in vivo pharmacokinetics and show nonspecific Fc-mediated sequestration in lymph nodes and the reticuloendothelial system. Aglycosylated IgG_1_ antibodies with abrogated Fc–FcγR binding yield lesser nonspecific uptake of the antibody and related radiocatabolites in vivo, yielding stealth targeting vectors. S228P hinge-mutated IgG_4_ antibodies overcome in vivo FAE to yield a better radiopharmacologic profile by eliminating uptake of antibody and associated radioactivity in the kidneys.

**IMPLICATIONS FOR PATIENT CARE:** Using immuno-PET to characterize the in vivo pharmacokinetics and biodistribution to uncover potential mechanism of action or toxicity of engineered antibodies can yield better and safe antibody-based drugs to improve patient care.
